# A System of Surgical Anatomy. Part 1. On the Structure of the Groin, Pelvis, and Perineum, &c.

**Published:** 1822-09-01

**Authors:** 


### VIII.

*A System of Surgical Anatomy. Part I. On the Structure of the Groin, Pelvis, and Perineum, as connected with Inguinal and Femoral Hernia; Tying the Iliac Arteries; and the Operation of Lithotomy. Illustrated by nine copper-plate Engravings.* By WILLIAM ANDERSON, Licentiate of the Royal College of Surgeons in Edinburgh, and Lecturer on Surgical Anatomy in New York. Quarto, pp. 200, with nine coloured Plates. New York, 1822.

Tros, Tyriusve nobis, nullo discrimine agetur.

IN this country it must be allowed that, within the last twenty years great advances have been made in the operative part of our profession. This may be safely attributed, in the first place, to a more correct knowledge of the changes produced on the structure by morbid actions; and secondly, to a more attentive and accurate examination of those parts likely to become the seat of surgical disease, or the subject of operation.

The advantages of such investigations have been well insisted on in all the writings of that late excellent surgeon and anatomist Mr. John Bell; and the successful labours of such men as Sir Astley Cooper, Burns, and Colles, in the field of surgical anatomy, can leave no doubt as to the importance of the subject. The advantage of keeping in view the ultimate object of anatomical enquiry cannot be doubted; and Mr. Colles has aptly enough observed, that otherwise "the inexperienced student, taught to regard anatomy without any references to its uses, views it only as a collection of detached and uninteresting facts, and a catalogue of barbarous and unmeaning terms."

Our brethren of the new world have not been inattentive to this most interesting and important study, and, in consequence, many of their names adorn the records of surgery.

Mr. Anderson, it appears, has made this branch a particular object of his attention, and has been engaged, not unsuccessfully, as we may gather from the work before us, in communicating his knowledge to others. We have no doubt that, by his labours, he will, ere long, reap a rich and ample harvest. While we are thus inclined to approve of the praiseworthy intentions of the author, and generally to applaud the execution of the task, we know that he would not thank us for withholding what remarks we may have made on some particular points; nor could we reconcile such a course to the duty we owe our brethren at large.

Our author has, in this volume, embraced the surgical anatomy of three most important operations—those for hernia, aneurism at the top of the thigh, and stone in the urinary bladder.

The anatomy of hernia has lately occupied the attention of the most eminent surgeons, as Cooper, Scarpa, Hey, &c. yet, notwithstanding the labours of these, and many others high in the profession, it remains still one of the most puzzling and confused subjects to the surgical pupil. This has arisen, we are convinced, not so much from the complicated nature of the structure, as from the confusion of terms and the endless differences in the descriptions; there is such a jumble of tendons and ligaments, straight and crooked, and sheaths and fascias, and arches, with pillars and rings, that the pupil is perfectly confounded and lost amongst them. But this is not the worst of it, for we find many different names applied to the same part, or to its subdivisions; for instance, the femoral ligament, the falciform process of the fascia lata, and the sheath of the vessels, &c. are all names applied to the same structure. Were surgeons and anatomists, by common consent, to abandon such superfluity of terms, (for when a name, however absurd, is once bestowed, it is seldom forgotten, especially by those who *talk* about anatomy,) a great deal of difficulty would be done away with.

From the following annunciation in the preface, we are led to expect something new on the subject of hernia.

"Upon the structure connected with hernia I have introduced an innovation, having delineated and described a crural arch, distinct from the ligament of Poupart:—this I send forth with all becoming humility, submitting the matter to the investigation of the careful anatomist, and to those who may yet have to divide the structure of a femoral hernia in the living subject. The operations upon the iliac arteries I have considered, because I feel myself prepared to recommend *a more safe operation* for securing them with ligature than has been pursued." Preface, p. 8.*

Of the comparative safety and success of the operations on the iliacs hereafter; the innovations in the anatomy of

---

* We cannot exactly understand the tendency of the following observation in the preface. Speaking of his chapter on Lithotomy, our author remarks that he "might have introduced the subject by an essay on the doctrines of Bacon," &c. Perhaps it is, that such is the practice in some of the transatlantic schools, as well as in a few of those in our own happy country. We heartily agree with our author in condemning such affectation and conceit.

hernia we are bound, in justice to those concerned, to say are not quite *new ones*. The formation of the crural arch, and nearly the whole of Mr. Anderson's new anatomy was described and delineated by Mr. Liston of Edinburgh, in a memoir on the subject, published in 1819. The work is noticed by Mr. A. in page 62, but we are inclined to question if he has seen more than the title which he quotes. By this remark, we by no means wish to cast any reflection upon the respected author—but the reverse. Mr. Anderson's views of the structure are most correct, excepting only in a few points, and these of comparatively little practical importance. In the essential points, he possesses most accurate notions, and for the *discovery* of the innovations we give him full credit.

The anatomy of the groin, is preceded by some grave and rather minute directions as to the shaving the hair of the pubes, previous to operations, and the disagreeable and painful consequences of neglecting this precaution. The course of the iliac is then accurately marked, and "we can here," says he, "feel it pulsate in the living subject; and here, we can, by pressing it on the brim, restrain any sudden hæmorrhage, from the vessels of the lower extremity, until a *tourniquet* can be procured." 36.

Contrary to the dicta of the late Mr. John Bell, it is now well ascertained, that sufficient pressure can be made, not only to stop the pulsation, but also to arrest the flow of blood, in any artery, without having recourse to the usual contrivance. No complications of screws or rollers is at all necessary to suppress bleeding in an artery, however large. The knowledge of this fact has induced many surgeons, both in the public service and in civil life, to abandon entirely the use of the tourniquet in amputations, aneurisms, or wounded arteries. The advantages of such a proceeding are very great. The surgeon becomes more fearless and bold, in cases of difficulty, and his operations, for the same reason, are rendered more rapid, and, strange to say, more bloodless. The surgeon must be timorous indeed, who could think of using pressure by a tourniquet or otherwise, in aneurism, yet, from a fear of cutting into the vessel, we have seen the tourniquet applied tightly in this operation. The venous ramifications are thus gorged, and their bleeding obscures the incision. Their trunks are distended and conceal the artery which is sought;—but, worse than all, the operator cuts on a distorted limb, the muscles of which are squeezed together, and the relative position of the parts thus lost.* Thus, an opera-

---

* This, to be sure, is of little consequence to men, who know the parts in neither one state nor another.

tion, which ought to be finished, under any circumstances, in two or three minutes, is protracted for twenty minutes or half an hour, and sometimes a little longer. Such is the case in a great northern hospital, where perhaps operations are performed with more *deliberation* (John Bell has called it "cruel deliberation") than any where else on the face of the earth. But more of this anon. The disuse of the tourniquet in amputation is equally to be desired; but our observations on this subject we shall defer till another opportunity. We may only remark, that it appears strange that surgeons should still persist in the use of this instrument in the smaller amputations, when they find that those of the shoulder and hip-joint can be accomplished safely by slight pressure on the principal vessel.

Though Mr. Anderson has very properly drawn a distinction betwixt the ligament of Poupart and the crural arch, still he does not appear to have observed *all the connexions* of the fasciæ with the latter band of fibres. One passage only, in confirmation of this position, is selected. Speaking of the superficial fascia, "while trying to remove it," says he, "from Poupart's ligament, we are almost sure to cut it through, for, as has been already observed, it appears with it one substance."

A short statement of the views we have been led to adopt, from our own examinations of the structure, will enable us to explain completely the state of this matter, and bring us more satisfactorily to the conclusion of this part of the subject, than by following our author, and bestowing on the different passages that share of praise or blame they might demand.

The fascia lata of the thigh is divided into two portions, the iliac and pubal. The former passing above the large vessels of the thigh, (more superficially,) the other lining the muscles under them. The iliac portion presents, at the entrance of the saphæna vein into the trunk, a falciform edge, (the process of that name.) This iliac portion, as well as its falciform process, is divided into two layers as it approaches the lower boundary of the abdominal muscles, the ligament of Poupart. These layers receive the ligament and muscles betwixt them.—The one layer (in other words superficial abdominal fascia) lies immediately under the common integuments, and covers the tendon of the external oblique. The deeper layer (fascia transversalis) lines, first of all, the inner surface of the internal oblique, and above that the transversalis muscles. At the separation of these two sheaths, a strong fibrous band passes across the space betwixt the anterior superior spinous process of the ileum, to the tuberosity and spine of the pubis. This band is slightly connected, in some subjects, by cellular substance to the ligamant of Poupart; in others, more firmly. In none is the superficial fascia firmly attached to the ligament. If an attempt is made to dissect this sheath much lower than the ligament, its connexion with the fascia lata will inevitably be cut. In all subjects the ligament of Poupart can be completely removed without diminishing at all the tightness of the opening through which femoral hernia passes. That opening is situated betwixt the inner edge of that band of fibres (which we perfectly agree with Mr. Anderson in styling the crural arch) and the femoral vein. We need not observe as to the passage of the chord through the fascia transversalis, excepting to recommend Mr. A. to look into *Cloquet* "*sur les Hernies, &c.*" where he will find a most accurate description and *delineation* too of the covering which the spermatic chord receives from the fascia transversalis.

The extent of the superficial fascia is well described in the volume under consideration.

We left the pubal portion of the fascia lata under the vessels. It is continued upwards over the brim of the pelvis, and becomes continuous with the strong covering of the iliac and psoas muscles. A great deal is said in this as well as other works of the *sheath of the vessels* in this situation. To us it appears a vague, ambiguous, and improper term, likely to lead to false notions. The vessels throughout the body are provided with a dense cellular covering, which binds them and their accompanying nerves together. This is *properly their sheath*, and is common to all. Why the vessels in the groin should have two sheaths, is beyond our comprehension. The *proper sheath*, it is evident, can have nothing to do with hernia. But the *sheath* which is said to be concerned in this disease, is the posterior or deep layer of the falciform process, or that which is continuous with the fascia transversalis. In treating thus lightly of the *great* sheath of the vessels in the groin, we know that we differ from the highest surgical authority in Great Britain—no less than that of Sir Astley Cooper. But we do so with all due deference and respect. After all, it is only as to names; and our reason for differing at all is, that we are of opinion that the vessels, in this situation, have no more title to two sheaths, than those in other parts of the body of equal or greater importance.

The passage of the chord is accurately and beautifully described by Mr. Anderson, more especially the alteration which takes place in the inguinal canal, during the growth of the young subject. On this subject he has borrowed freely, and we think judiciously, from that most accurate anatomist, the late Mr. Allan Burns. The inguinal canal is known to be quite direct in the fœtus at birth; it gradually becomes oblique, and we might say *valvular*, so as to support the bowels and obviate protrusion. So it is that congenital hernia or inguinal hernia, occurring in young subjects, may be completely got the better of by proper care; whereas, this event is seldom to be looked for in the adult.

We have heard of the application of oak bark *(splashed)** over the abdominal apertures, with a view of promoting their contraction. We should be inclined to trust to this practice as much as to the use of warm fomentations to relax the same parts, in cases of strangulation. Who, that ever saw the unyielding tendinous openings, could rationally think of either the one practice or the other? The plan savours much of that school in the North, where it is inculcated that *exuberant callus* should and can be repressed in the fractured clavicles of young ladies by glued pasteboard, and other such contrivances.†

On the formation of the ligament of Gimbernat, more properly the crescentic portion of the crural arch, independently of Poupart's ligament; and the crossing of the fibres of the recti to strengthen that part, it is unnecessary to enter. We refer to the plates and description in the memoir we have already quoted.‡ The above descriptive sketch may to some appear useless and tiresome. We have paid a good deal of attention to the anatomy of this region, and it will be seen that we are not of those who consider a knowledge of the fasciæ unnecessary to the pathologist and operating surgeon. It is only from a correct acquaintance with these parts, and their connexions, that we can understand the modifications of hernial tumours, or proceed a single step with safety in any operation on the living body. This mode of considering the parts in a connected view, is the only one by which we are led to understand at all the effects of position in facilitating the reduction of hernia by the taxis or otherwise.

Mr. A. seems to extend the term superficial fasciæ to the

---

* Mr. Lizars. Edinb. Med. and Surg. Journal, No. LXXII.

† Did those who talk thus wildly of exuberant callus ever read Pott or Bell (NOT Benjamin) or did they ever see a fracture well attended to? We suspect not.

‡ Memoir on the formation and connexions of the crural arch, &c. &c. by Robert Liston, Surgeon and Lecturer on Anatomy and Surgery. Edinb. 1819.

cellular covering of the whole body, and is hence led to speak of that layer on the fore and upper part of the thigh. The cellular substance in that situation, containing the glands of the groin, has little claim to such consideration. In our apprehension, the extension of the term must lead to great confusion. One more remark on this part of the subject, and we have done. In page 76 the following passage occurs :—

"Since that period, from my own observation, I am firmly of the belief, that the epigastric or external iliac artery is the more common source of the origin of the obturator. I think, notwithstanding, in the majority of these instances, a femoral hernia is more likely to lay upon the artery than be insinuated between this vessel and the brim of the pelvis in its way under the crural arch."

In this and the preceding remarks we most perfectly concur. We have examined cases of femoral hernia, in which the obturator passed off by a common trunk an inch and a half long with the epigastric; still that vessel surrounded *not* the neck of the sac, but crept behind it. Correct surgical rules can with difficulty be drawn from one old musty dry preparation in the collection of Dr. Barclay—the only instance, we believe, in which the obturator has been found to surroud the neck of the sac; whether it ran close to the crescentic portion of the arch or not, cannot now be ascertained. Mr. Hey thought that he could have divided the stricture safely, notwithstanding such a distribution, and so may any one who feels before he cuts, and knows what he should cut.

For a more full explanation of the separated state of the spermatic chord in large inguinal hernia, as also in hydrocele, we would refer to Scarpa's excellent work.* His observations on the mode of avoiding the vas deferens and vessels in the different operations are also worthy of attention. Speaking of large herniæ, which, by the bye, our author has said but little of, we may be allowed to make one observation—as to the great hazard of opening such tumours to their whole extent. The folly and danger, we had almost said crime, of such a proceeding, are well shown by some notes of a case we took down from a surgical lecture some years ago. The journals of a certain hospital in the North, *famous* for its operations, will, we believe, afford further particulars.

A middle aged man presented himself on account of a

---

* Scarpa on Hernia. Translated by John Henry Wishart, President of the Royal College of Surgeons of Edinburgh.

large scrotal hernia, 23 inches in circumference. It had been strangulated for eight days. He had stercoraceous vomiting and great pain of the sac, but none of the belly on any degree of pressure. *The tumour was opened, from one end to the other.* The intestines were found thickened and mortified in many parts, but adhering by coagulable lymph to the neck of the sac, and thus cut off from the peritoneal cavity. Attempts were made at reduction for an hour and a half, with but little effect. The patient was carried to bed and died in about an hour. On dissection, it appeared that of half of the small intestines contained in the sac, only about seven inches had been reduced. The adhesions to the neck of the sac were separated; and the upper part, completely torn across and presenting an open mouth, had poured its contents into the cavity of the peritoneum amongst the comparatively healthy intestines. These were covered with an almost imperceptible blush of red vessels.

It must be evident to the merest tyro in surgery, that in this case the inflammation was entirely confined to the gut in the sac, that from the duration of the strangulated state there was every reason to expect disorganization of the contents, and that though the gut *had been sound*, there was no chance of its being pressed into the abdominal cavity, contracted and unaccustomed as it had been to the presence of such a mass. *The attempt, so far successful, to reduce a mortified bowel*, must fill every feeling mind with horror and indignation. Nature might have done a great deal for this poor man, had the sac been slightly opened, and free passage afforded for the contents of the bowels. As it was, he had no chance.

Any person who had thought for himself, (those concerned might be incapable of such exertion,) or had looked into Scarpa's judicious remarks on the subject, could not have been guilty of such blundering. Far less is it possible to suppose that the learned translator of that eminent surgeon's works could have been witness of such a proceeding.

Of Mr. Anderson's diagnosis of hernia, we approve most highly. Every surgeon in extensive practice must meet with many such mistakes as are mentioned, both on the part of the patients and ignorant advisers. We have again and again seen buboes cut for hernia, and hernia for buboes; cirsocele treated with trusses, and so on; in one day we witnessed two such cases. The patients were in excruciatiug agony, from the great distention of the tumour. We have seen aneurisms poulticed, and blistered, and opened, and have even heard of the application of the actual cautery, in the form of moxa, to their surface. These things have happened, and must continue to happen, in the hands of those who will not take the trouble, or who are incapable of acquiring the fundamental branches of the profession.

No pupil of Mr. Anderson's, we will venture to predict, will have himself to blame for any such mistakes.

As to the operation, we have but one or two remarks to make, and the first is in condemnation of the instrument called a *directory*. Mr. A. is too good an anatomist to require such an instrument himself, and he should leave it as a distinguishing mark of those who grope on, uncertain how many layers (whether twenty or one) they require to divide before coming to the sac. Many a time have we witnessed fully twenty minutes occupied in splitting *curiously*, with a sharp pointed directory, the various layers over a hernial tumor. Such a plan can serve only to teaze and exhaust the patient, it cannot add to his safety. Scarpa talks of the hand unsupported in such incisions. It is the speediest and safest mode on the whole. The dissecting forceps will answer in raising the deepest layers if necessary. The danger of wounding the epigastric artery we consider not worth thinking of. If the incision is only carried so far as to relieve the stricture, it never can be wounded, whatever the direction may be. If the surgeon is determined to cut up the belly without an object, he may, *at the least*, encounter this artery. That it never was, by any chance, *unavoidably* cut in this operation, we are quite convinced.

"The epigastric artery," says Mr. John Bell, "never was cut—never will be cut—never can be cut. Various are the surgeons, good and bad, in country and in town, who have sought for the same, with their fingers some, and some with their knives; yet hath it not been found: It lieth at the back of almost every rupture, both of the groin and of the thigh, nor hath it been found playing bo-peep with the hernia;—as most notoriously the femoral artery hath been *detected*, so amusing itself, to the great terror of all beholders, and indescribable danger of the king's lieges."

We concur most heartily in our author's recommendation to proceed *instanter* to operation, whenever a gentle trial of the taxis has failed.

"Was I the subject," Mr. Anderson remarks, "of a strangulated hernia, I think I would not, under the most favorable circumstances, suffer the operation by the knife to be delayed more than six hours from the time of the first symptom of incarceration; a very small portion of which period, I would consent to have occupied with the taxis."

Much mischief may arise in a very few hours, more especially in femoral hernia, owing to the extreme tightness* of the stricture, and we should scarcely be inclined to grant even so long a delay as our author. We have been in the habit, of late, of proceeding to the operation *immediately* on being called, and have seen ample reason, in the success attending the practice, to continue in the same course.

We now come to that part of the work which treats of the anatomy and operations on the iliac arteries, &c.

The diseases and injuries of the arteries, and the operations required to remedy such, are of the utmost interest to the surgical practitioner; whether we regard the danger to the patient, the comparative simplicity of the proceedings necessary, or the beautiful, and, in general, successful efforts of nature during the process.

The splendid improvements of John Hunter on the old and barbarous *cure* for aneurism; (if cure it might be called, when scarce a patient was saved, and some of the most eminent surgeons preferred amputation, *in this disease* a most hazardous and desperate remedy,) the great operations of John Bell, Sir A. Cooper, and Mr. Abernethy; and the important pathological expositions of these eminent surgeons, as also of Jones and Scarpa, have reduced the treatment of this disease to a degree of certainty, almost unknown in any other. Operations on the great arteries, the bare mention of which would have appalled our forefathers, are now frequently put in practice. What would *they* have thought of Mr. Anderson's directions for the ligature of the aorta? This operation has indeed been accomplished by Sir A. Cooper, but it is not for those possessed of ordinary hands or heads, to imitate him in this. We shall confine our remarks to an operation more frequently required, and, by which, more lives are likely to be saved—the ligature of the external iliac artery.

> "The plan which I shall propose," says Mr. Anderson, "to enable the surgeon to get at these vessels in the living subject, will, I think, be accepted by all who will take the trouble to reflect upon the structure of the parts." 142.

The only unfavorable consequences which our author seems to apprehend, are inflammation of the peritoneum, and the occurrence of hernial protrusions. His directions are there-

---

* After dividing the stricture of femoral hernia and reducing the contents, we have, in some cases, found it almost impossible to insinuate the point of the ring finger under the crural arch.—How small then must it be previously, and how slight the scratch necessary to allow the return of the parts.

fore intended to obviate these accidents; they are to this purpose—to disturb the peritoneum as little as possible, and to make the incisions in the course of the muscular fibres, and no farther than absolutely necessary. Both good rules enough; the former, it is to be hoped, acted up to by all who have attempted the operation. We cannot approve of the mode of dissecting the integuments from the external oblique to such an extent (or to any extent at all) as Mr. A. proposes; nor can we see the purpose of it. In debilitated patients, or those advanced in life, such a practice would probably be followed by the sloughing both of the skin and tendon.* Surgeons differ much as to the direction of the in-incision. That recommended by Mr. Anderson certainly affords many advantages, and is approved of by good surgeons in this country. Mr. Abernethy's mode of incision admits of the artery being tied further from the seat of the disease, than either Sir Astley Cooper's or Mr. Anderson's; but then, the abdominal contents are left, in a measure, unsupported. A middle course might, perhaps, be followed with advantage. An incision might be made nearly parallel to the linea semilunaris, on the outer side of the inguinal canal, by which very few of the fibres of the external oblique, or indeed of the internal, at this point, will be divided, and at the same time, the artery reached an inch and a half or two inches above the ligament of Poupart.† By pursuing this method, we should have little dread either of inflammation or hernia. What has always given us the greatest uneasiness, after the ligature of an artery, is the risk of secondary hæmorrhage; and to the prevention of this accident, all our endeavours, both in the performance of the operation, and in our after treatment, ought to be most anxiously directed.‡

It might be considered somewhat out of place for us to enter here, into the question regarding the propriety of applying the ligature, so as to divide or not, the inner coats of an

---

* Such is occasionally the result of the extensive separation of the skin in amputation; as we have seen practised. When sloughing occurs, there being no other covering, the bone, of course, protrudes, and the patient sinks under a complication of affliction, or drags out a miserable existence with conical and irritable stump.

† This plan appears to have been followed by Mr. Liston, of Edinburgh.—*Ed. Med. and Surg. Journal, No.* LXII. *page* 72.

‡ We cannot approve of Mr. Anderson's observation, that on the introduction of the aneurism needle, "the artery will now be elevated through the ring." This could not be done without separating the vessel from its connexions, and thus endangering the death or ulceration of its coats.

artery. It is well ascertained, that the approximation of the inner surface is sufficient to incite such an action, as will terminate in effusion of lymph and obliteration of the calibre of a vessel. The presence of a foreign body, as a ligature applied slackly to the coats of a vessel, will, now and then, produce the same effect. This may be all very well in the way of experiment, but we have long been firmly convinced, that the breach of continuity in the internal coats of an artery, and their close coaptation *insure*, more completely, the effusion of lymph and obliteration of its canal. If a small, round ligature is applied, without the intervention of nerve, sheath, or other part, to an artery, and so tightly as to divide the inner coats, it will separate readily in ten, twelve, or sixteen days, and leave no reason to regret the *non-employment* of pincers—presse-arteres—or temporary ligatures. In short, we are advocates for the single permanent ligature *properly applied.* What we mean by properly applied may be asked. The sheath must be cut upon, exposed, and opened slightly, with the point of the knife; the artery is then to be surrounded by a small, round, and firm, silk ligature, conveyed by means of a very fine aneurism needle, of a slight curvature—a single knot is then to be drawn tightly, and secured by a second;—the greatest care being taken, both in applying the ligature and tightening it, to leave the artery *undisturbed.**

Mr. Anderson observes, "when these operations are attempted for the cure of aneurism, there is no sufficient reason, generally speaking, why they should prove fatal. Yet such has been the case and may occur again, *if an unrestrained use of the knife* is resorted to." P. 142.

We are willing to admit readily, that the knife is not to be used entirely in the operation for inguinal aneurism, in separating the peritoneum from the abdominal parietes. Mr. Anderson, we are convinced, does not mean to reprobate the free use of the knife in *all* operations for aneurism, or to recommend the barbarous, unsurgical, we would almost say *inhuman* mode of getting to an artery, by tearing with the fingers, handle of a knife, or other blunt implement, as recommended and practised by some surgeons. This can only

---

* We have frequently heard Mr. Liston, of Edinburgh, attribute his uniform success, in operations for aneurism, to such a mode of proceeding as we have described—the clean incisions down to the vessel, and the inclusion of it in a small ligature without disturbing or separating it at all from its connexions. He has, we understand, again and again, tied all the arteries of the body, amongst the rest, the *left* subclavian, without losing a patient, and without the occurrence of secondary bleeding, or any untoward accident.

proceed from the operator being conscious of his deficiency in anatomical knowledge. He cannot say that he will make his way more quickly or easily to the artery, with a blunt than with a sharp instrument; though he may, at the instant, do it with more safety to his own reputation. But what is the reputation of such a practitioner to the *patient's welfare?* No one will be so bold as to say that *he* will be saved less pain at the time, or that *his* chance of recovery will be the greater for such a mode of operating. The cellular texture, treated in the fashion we have described above, cannot adhere so as to support the vessel—it being well known *that every lacerated wound must suppurate* more or less. The artery torn up from its bed must die to a greater or less extent, and secondary hæmorrhage be the result. The consequence of such a proceeding is well exemplified in the following case which occurred lately in a large hospital.

A stout man, 50 years of age, was admitted on account of popliteal aneurism of two years standing. The tumour was of a moderate size, and the pulsation distinct. A professor of anatomy was employed to make a survey* of the parts, and when the vessel *was found* to mark its course† by three points of caustic, thus * * *. Notwithstanding these precautions, a quarter of an hour was spent, next day, in seeking both inside and outside of the thigh, for the vessel. When it was found, it was torn and separated from its bed by the introduction of the fingers of the operator and assistants under it. A large, coarse, flat, and uneven ligature, of four threads, was then tied close to the lower attachments of the artery, leaving at least an inch of its coats insulated betwixt that and the heart. As was to be anticipated, bleeding came on, about the fifteenth day, early in the evening. The hæmorrhage, which was profuse, was suppressed by a compress and bandage, and the surgeon sent for express. He declined attending. In the morning, when required in consequence of a recurrence of the same accident, he again refused. At the visit *he ordered twenty ounces of blood to*

---

* By drawing lines and raising angles!—a contrivance to make up for deficiency in relative anatomy—a regular trigonometrical survey.

† It is not to be supposed that a person filling the honourable situation of surgeon to a large public hospital, should be under the necessity of employing another, probably equally ignorant as himself, to mark out the situation of the femoral artery, previous to the operation for aneurism. This expedient would, no doubt, convey to our American readers, a very favourable opinion of the state of surgery in some parts of this country.

*be drawn from the arm.** That evening the hæmorrhage again appeared, and ceased spontaneously. The patient expressed great anxiety that something should be done to save his life, and that, at all events, the person who had operated should visit him. The attendants were equally desirous of assistance, and an express was again sent but in vain. Next morning, after a very urgent message, the operator appeared. Symptoms of mortification had shewn themselves, and the patient, then moribund, lingered till the evening—apparently destroyed by the operation and its consequences, without an effort being made to repair the defects or effects of either.

The examination shewed the limb immensely swollen and livid—the cicatrix sphacelated—the separation of the femoral artery for a great extent above the ligature—this portion imbedded in suppurating cellular substance, and perforated by numerous sloughy holes—a large false aneurism in the thigh, containing at least three pounds of coagulated blood—abundant effusion of lympth below the ligature, none above; and lastly, the perfect soundness of the whole vessel from the bifurcation of the aorta to the tumour, excepting at the very place of rupture in the ham, where there was even less appearance of degeneration in the coats than might have been expected—only a couple of slight specks. The artery was most limber and sound, its coats below the ligature, especially the muscular, being thickened by long continued exertion.

This case requires no commentary. Let those who are capable judge if the operation was performed in accordance with the best rules of modern surgery or not. Certain it is that after the occurrence of the hæmorrhage, the only chance which the poor man had, viz. the ligature of the iliac was withheld. This chance the surgeon was bound to give, and, in all probability, success would have followed the proper performance of this operation.

We regret the fatal result of Mr. Anderson's two cases in which he was called upon to tie the external iliac. The operations appear to have been resorted to under very unfavourable circumstances. He would have gratified us, however, by stating whether peritoneal inflammation or secondary bleeding was the cause of death.

---

* We should be glad to receive an explanation of what was expected from this extraordinary practice. It would appear that violent bleeding from a ruptured femoral artery had been confounded with the case of oozing from the mucous surface, in which venesection is now and then practised, on what principle, or with what propriety, we cannot pretend to say.

We shall now enter shortly on the last subject treated of in this respectable work, and we have the satisfaction of conveying to Mr. Anderson our most unqualified approbation of his anatomy of the parts concerned in the operation of lithotomy. His description of the iliac fascia and its connexions forming the partition in the pelvis, is most accurate. We question, however, the propriety of dividing so much of the side of the bladder, and we are advocates for the employment of one cutting instrument only, in making our way into that viscus. We are aware that an experienced and bold surgeon and anatomist will find his way, and that with perfect safety to his patient, put what instrument you will into his hands, be it knife, gorget, or lithotome. The staff grooved *betwixt the side and convex part*, as recommended by Mr. Anderson, is employed by some eminent surgeons in this country;—we may notice one of them, Mr. Charles Bell. We would recommend Mr. A. to look into what has been done in the school, of which that able surgeon is the the head, in regard to the anatomy of the bladder and parts connected with it. A few additional remarks on this interesting subject will be excused—and first, in regard to the direction of the wound.

The incisions ought evidently to be free, and the direction of the wound dependent. The external incision (as every external incision ought to be made in reference to the object in view) must be extensive,* and carried completely beyond the verge of the anus.

The fascia of the perineum, transverse muscle, and levator

---

* The division of an inch or two of integuments, more or less, in any operation, will neither add to the patient's suffering, nor retard the cure one day—whilst room will be afforded for the speedy removal of the disease. On the contrary, the division of too little integuments, by but half an inch, will render the ready accomplishment of the after-parts of an operation most difficult, tedious, and, from the delay, even dangerous to a patient. We have seen many patients die exhausted, before the first dressing from the protraction of operations, in themselves of no great importance. From the teachers who, in their anxiety to conceal the blunders of themselves and brethren, and attribute such unfortunate results to any thing but the true cause, we differ most widely. *Inflammation* will not always account for a patient's death; nor will *all* the young men attending a public hospital be cajoled into such a belief, the more especially, when neither distended vessels nor effused lymph can be discovered. We would, if we may be allowed, recommend to such people a little more candour. Let them, at least now and then, acknowledge their errors; and when they have occasion to speak of adhesions and preternatural sacs, they will perhaps be more readily credited. Two or three inches of the femur sticking through an unhealthy stump is certainly more likely to occasion death than membranous adhesions of the lungs, perhaps of twenty years standing.

ani, must be freely divided, and all with a view of allowing the easy extraction of the stone, and ready escape of the urine. It strikes us to be of less consequence how much or how little of the neck of the bladder is wounded, provided that no force is necessary in the extraction, and that the urine has a free exit through the external wound; *if it has*, extravasation will not take place; *if it has not*, whatever may be the extent of the wound, or whatever fascia is cut or not cut, extravasated it must be; and the almost inevitable consequence is the destruction of the patient. We have had the misfortune to witness the death of a good many patients after this operation. Those of them who lived over twenty-four hours, and have been attentively examined after death, have uniformly presented most unequivocal marks of urinary infiltration into the cellular substance about the neck of the bladder. It is a difficult matter, however, to convince some practitioners of such a circumstance as this, and more particularly when they have been long engaged in the practice of representing softening of the kidnies, thickening of the bladder, or old membranous adhesions of the lungs, as causes of death in such cases. We have been almost tempted to apply the infiltrated cellular substance to the olfactory organs of these sceptics, thus giving them to understand that if they did not see, they might, at least, smell the cause of death in their patients. Such was the coarse, though impressive way, in which the late Dr. Monro, of Edinburgh, is said to have conveyed a rebuke to a practitioner (surgeon he could scarcely be called) who had perseveringly mistaken a case of ruptured urethra and consequent extravation, for some affection of the testicles. The Dr. found the patient in *articulo mortis*, evidently from the culpable ignorance of the attendant; and, in his indignation, applied the cloth with which the scrotum had been enveloped, soaked as it was with urine, in the way, and with the observation, we have above mentioned.

In the fatal lithotomy cases, we have, in general, noticed that the direction of the forceps in search of the stone was downwards and backwards, instead of inclining rather upwards. What can possibly occur, when, in addition to its improper direction, the wound is crammed with lint, and the patient's thighs bound firmly together? The passage of the urine through the wound being denied, a little may find its way through the urethra; the remainder insinuates itself by the side of the bladder; pain comes on with great restlessness; peritoneal inflammation is suspected; the patient is bled copiously and repeatedly;—leeched, blistered, and dies. When the wound is made, as we have already described, and the free exit of the urine perhaps still farther secured by the use of an elastic gum tube, no such results are likely to happen.

The internal pudic artery, we apprehend, is in greater danger when the deep incisions are made with a beaked instrument of whatever kind, pushed, as it must be, along the groove of the staff, than when a sharp pointed one is made use of. The incisions, in the one case, are made in a measure at random, and towards the ramus of the ischium. In the other mode, the fore-finger of the left hand guides the knife, and the incisions may even be made *from* the seat of the vessel. We have seen this artery cut, and should not chuse to witness such another accident. The blunder is more easily committed than remedied. Mr. Thomas Blizard, than whom no surgeon could be more bold or dexterous, was luckily present, and succeeded in arresting the hæmorrhage by ligature.

Another accident, equally serious, we have still to notice, as now and then occurring in this operation—the wounding of the rectum. The circumstance of one of the greatest and most successful lithotomists* this country ever produced, having twice committed this mistake in attempting a most hazardous mode of opening the bladder, ought not to be admitted as an excuse, now that the principles on which the operation is to be conducted, are more fully understood. The wound of the rectum can arise only from the most culpable ignorance or inattention, and is an accident which almost inevitably will render the patient miserable for the remainder of his life. Life, in fact, cannot be long protracted under such suffering and misery, nor is it much to be desired. By the constant draining of the urine into the rectum, and the passage of flatus and liquid fæces through the urethra, the healing of the wound is effectually prevented. Some means might perhaps be employed to promote this most desirable object, before the opening becomes fistulous. In such cases as we have met with, the opening had existed for many months, (God forbid that we should meet with them at an earlier period, or at any period) and proved incurable—notwithstanding every attempt, and some of them bold enough. One case was that of a gentleman of some consequence. The operation was most tedious, and in the evening the urine, fæces, and flatus, passed as we have abovementioned. The wound was opened repeatedly, and the patient tormented with the forceps in the bladder in search of fragments of

* Cheselden.

stone, until he was exhausted. He dragged out a miserable existence for a few years, unable, of course, to go into company, and incapable of any enjoyment. We have seen and heard of similar instances both in *public* and private. It is said that such an accident is not fatal—it may not be necessarily so, but the consequence of it are sufficiently disastrous to the patient. Nor can the consciousness of having committed such a horrible blunder be at all supportable by the surgeon, unless he be entirely devoid of feeling, and lost to shame. We may be told, in answer to all this, that some surgeons cut the rectum intentionally in this operation. Out of eleven cases on record of this new recto-vesical operation, as it is called, we find that two died and other two continued to have a communication betwixt the bladder and rectum. These facts are sufficient to prevent us from attempting or recommending such a practice.

What the advantages are to be derived from laying the rectum and bladder into one, we could never discover; whether that is done by accident or design. The only reason at all plausible is, that in this way, a larger stone may be extracted than by the lateral incision. We will venture to assert, and without fear of contradiction, that if the rectum be completely emptied, and the incision carried well down by its side, an opening large enough for the extraction of any stone that will pass the *outlet* of the pelvis, can be made. We do not apprehend that either the recto-vesical or old high operation, with all its new complications, will ever come into vogue in this country; the more especially, when we consider the great success attendant on the lateral method in the hands of those qualified to perform it. Incapable men have had, and have still the presumption, to attempt this and other operations which can only be safely accomplished by those who understand the structure of the parts, and have hands to execute what the head directs. No mode of operation and no form of instrument can insure success in any operation, or make up to such clumsy operators for their lack of knowledge, their wavering of mind, or trembling of hand. Out of fifty-two patients operated on by the lateral method, Cheselden lost only two. Mr. Barlow, of Blackburn, out of more than sixty, has lost also two; whilst Mr. Martineau, of Norwich, has, by most extraordinary success, saved eighty-two out of eighty-four. In a Royal Infirmary of the North, we witnessed, during last winter, three operations for stone. Out of the three, one escaped.*

---

* Such is likely to be the success, not in this alone, but in all other

It is for such unsuccessful practitioners to contrive new modes of operation, or rather, in charity, to abandon operations entirely. The latter course would be the most conscientious and advisable, in whatever way we view it—whether for *their own interest*, or that of suffering humanity. We have put *first* the stronger incentive to a right mode of action.

We doubt if Mr. Anderson's book will be either understood or properly appreciated by such people. Be this as it may, we shall, however, extract one good advice from his preface, for their edification.

"By habitual dissection, an easy use of the knife is acquired, so much looked for in an operator, by which the parts are cut with more *safety* to the patient, and *satisfaction* to those around. An operating surgeon, therefore, should be a clean dissector; as most operations consist in neatly dividing and separating living parts. The student should, on this account, thus qualify himself, and with accuracy, in the dead subject, that by frequent employment therein, he may become so familiar with the general structure of parts, that he will not be confused or interrupted by the flowing of blood during an operation; as this will be more or less an impediment as the surgeon's anatomical knowledge is deficient. *Moreover, as he is frequently called to cut within a hair's breadth of the patient's life, it is highly necessary indeed that he knows where he treads.*"

Some forms of expression in the work before us, we confess, sounded rather strange in our ears; but we believe them to be not peculiar to Mr. Anderson alone, but to the whole of our transatlantic brethren. The plates are, in general, well executed, and creditable enough to the artists employed. The rectum, vesiculæ seminales, and prostate, in the last, have but a distant resemblance to any thing in the pelvis, which we have seen in this country.

We now take leave of our author, by assuring him of the pleasure we have had in the perusal of this part of his work. He has already given proofs that he is an industrious anatomist and an accurate observer of nature. We hope he will pursue the subject in the second part with vigour, as we are firmly convinced that the undertaking is well calculated to advance the science of surgical anatomy in that quarter of the world.

---

operations, so long as surgeons require political judges and hired pleaders to support and defend the result of their practice.

